# Fractional CO_2_ laser therapy for genitourinary syndrome of menopause: symptom-specific trajectories, exposure–outcome associations, and ultrasonographic changes in vulvar soft tissue in a cohort of 826 women

**DOI:** 10.3389/frph.2026.1776174

**Published:** 2026-03-04

**Authors:** Mariko Hatta, Hiroaki Ohta, Kuniaki Ota, Remi Yoshikata, Stefano Salvatore

**Affiliations:** 1 Juno-Vesta Clinic Hatta, Chiba, Japan; 2 Department of Obstetrics and Gynecology, Kawasaki Medical School, Okayama, Japan; 3 BASEGATE Yokohama Kannai Clinic, Yokohama, Japan; 4 Obstetrics and Gynecology Unit, Vita-Salute San Raffaele University, IRCCS San Raffaele Hospital, Urogynecology Unit, Milan, Italy

**Keywords:** dyspareunia, fractional CO_2_ laser, genitourinary syndrome of menopause, symptom trajectories, treatment exposure, ultrasonogaphy, vaginal laser therapy, vulvar soft tissue

## Abstract

**Background:**

Genitourinary syndrome of menopause (GSM) is a chronic condition that impairs quality of life and sexual function. Fractional CO_2_ laser therapy is a non-hormonal option, but large real-world data on symptom trajectories, durability, and ultrasonographic vulvar changes are limited. We evaluated symptom trajectories, responder rates, exposure–outcome associations, and vulvar tissue changes in a clinical cohort.

**Methods:**

We conducted a retrospective observational study at a single clinic in Japan. From 2016 to 2023, 826 women underwent fractional CO₂ vaginal and vulvar laser therapy (2,129 sessions). Symptoms were assessed using VAS (0–10) scores for six domains. Short-term outcomes were evaluated 20–59 days after the first session (*n* = 327), and long-term outcomes 10–14 months after the final session (*n* = 94). Responders were defined as a ≥2-point VAS improvement among women with baseline VAS ≥2.

**Objective:**

outcomes included ultrasonographic labia majora thickness; post-treatment imaging corresponded to the same windows when paired measurements were available. Patient satisfaction and adverse events were recorded.

**Results:**

Mean age at first treatment was 61.9 ± 10.2 years (range, 29–87). All six symptoms improved short term, with the largest improvements typically in dyspareunia and vaginal dryness. At 10–14 months, improvements in dryness and urinary leakage attenuated, whereas dyspareunia was most durable. Labia majora thickness increased overall (16.9 ± 4.5–18.9 ± 3.1 mm), with thickening in 81.5% of women with paired measurements. Higher responder rates were observed among women receiving more sessions; however, these findings are associational and may reflect baseline severity and follow-up engagement. Satisfaction was high, and no serious adverse events were observed.

**Conclusions:**

In this real-world cohort, fractional CO_2_ vaginal and vulvar laser therapy for GSM was associated with reduced symptom severity and ultrasonographic thickening of the labia majora in a subset with paired measurements. Given the retrospective uncontrolled design, incomplete follow-up, and placebo effects in sham-controlled trials, findings should be interpreted as descriptive associations, not causal effects. Controlled studies are needed to confirm effectiveness, durability, and maintenance strategies.

## Introduction

Genitourinary syndrome of menopause (GSM) is a chronic condition caused by hypoestrogenism and characterized by vulvovaginal dryness, irritation, burning, dyspareunia, and lower urinary tract symptoms. These manifestations often persist and worsen with time and can substantially impair quality of life and sexual function in midlife and older women (1, 2). Recent expert statements have therefore emphasized GSM as a female-specific non-communicable disease (NCD) that warrants proactive, long-term management rather than being regarded as an inevitable consequence of aging (1).

Epidemiologic data from Japan indicate that GSM-related symptoms are highly prevalent not only after menopause but also in late reproductive and perimenopausal ages. Cross-sectional surveys have reported frequent vulvovaginal symptoms, including dryness, pruritus, and dyspareunia, among Japanese women in their 20s–40s (3), and GSM-related complaints in women in their 40s–60s have been strongly associated with reduced sexual activity and sexual dysfunction (4). GSM is common and often underrecognized, and many women report persistent symptoms and unmet needs despite available therapies (1, 5). In a nationwide web-based survey of 10,000 Japanese women aged 40–90 years, approximately 45% reported at least one genital or urinary symptom compatible with GSM, and many expressed dissatisfaction with available management options and persistent psychological distress (6, 7). These findings suggest that tens of millions of Japanese women may be affected by GSM and underscore the need for effective and acceptable treatment strategies in real-world practice (3, 4, 6, 7).

According to the 2020 position statement of The North American Menopause Society (NAMS), recommended management of GSM includes nonhormonal vaginal moisturizers and lubricants as initial treatment, with low-dose vaginal estrogens, intravaginal dehydroepiandrosterone (DHEA), and oral ospemifene as subsequent options in women without contraindications (1). However, in Japan many of these evidence-based local hormonal therapies are not commercially available because of drug lag and drug loss. As a result, Japanese women with GSM often rely on over-the-counter moisturizers, lubricants, probiotics, and off-label approaches, and their satisfaction with symptom relief remains limited (7). This therapeutic gap has fueled interest in non-hormonal, device-based therapies such as vaginal and vulvar fractional carbon dioxide (CO_2_) laser.

Fractional CO_2_ laser is an energy-based technology that creates microscopic columns of controlled thermal injury in the epithelium and superficial stroma, thereby triggering wound-healing responses that promote collagen remodeling, neoangiogenesis, and restoration of tissue elasticity and lubrication (8). Early prospective studies suggested that vaginal fractional CO_2_ laser improves vulvovaginal atrophy and GSM symptoms, particularly dryness and dyspareunia, in women who are unable or unwilling to use local estrogen therapy (9, 10). More recently, observational cohorts and clinical trials have reported short-term improvements in multiple symptom domains; however, randomized controlled trials comparing fractional CO_2_ laser with sham treatment or standard hormonal therapy have yielded mixed results, and systematic reviews and meta-analyses restricted to randomized trials have concluded that the certainty of evidence is low (11–13). Professional societies have therefore adopted a cautious stance: the Royal College of Obstetricians and Gynaecologists (RCOG) has stated that vaginal laser therapy for GSM should not be routinely offered outside clinical trials (14), and editorial commentaries have raised concerns about potential harms and overuse in the absence of robust data (15).

Despite these uncertainties, use of fractional CO_2_ laser for GSM has expanded in daily practice, especially in settings where access to approved local hormonal treatments is limited or where patients prefer to avoid hormones. In Japan, vaginal and vulvar laser therapy is increasingly offered as a self-pay, non-hormonal option in gynecologic clinics; however, large real-world datasets describing symptom trajectories, durability of treatment effects, objective changes in vulvar and vaginal tissue characteristics, and the influence of concomitant systemic hormone replacement therapy (HRT) are scarce. Therefore, the present study aimed to describe symptom-specific trajectories, responder rates, and safety outcomes of combined vaginal and vulvar fractional CO_2_ laser therapy for GSM in a large single-center cohort. Any comparisons according to concomitant systemic HRT were planned as exploratory analyses rather than causal comparative effectiveness evaluations.

## Materials and methods

### Study design and participants

**Figure 1:**
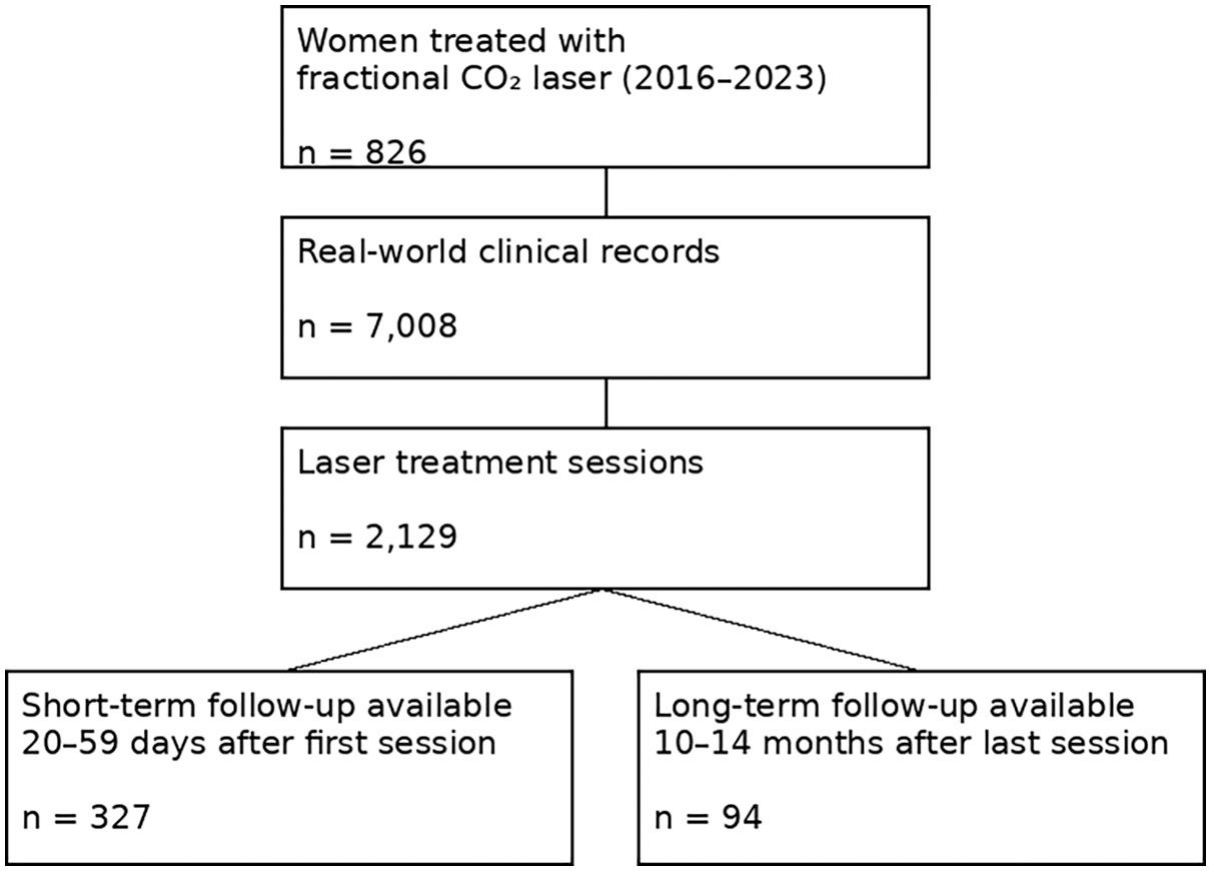
Study flow and availability of follow-up windows. A schematic summary of the clinical cohort, total records, laser treatment sessions, and the number of women with evaluable short-term (20–59 days after first session) and long-term (10–14 months after last session) follow-up records.

This retrospective observational study was conducted at Juno Vesta Clinic Hatta (Matsudo, Chiba, Japan). Over an 8-year period from January 2016 to December 2023, fractional CO_2_ vaginal and vulvar laser therapy was performed in 2,129 sessions among 826 women who requested treatment and provided written informed consent. The dataset comprised 7,008 real-world clinical records. At the time of the first treatment, the mean age was 61.9 ± 10.2 years (range, 29–87 years). This value corresponds to age at first laser session in the final analytic dataset and is used consistently throughout the manuscript. Follow-up outcomes were evaluated using pre-specified time windows based on the timing of questionnaire completion relative to the first and final laser sessions (Figure 1).

### Laser therapy protocol

Before treatment, all patients were examined to exclude contraindications such as vaginal or vulvar inflammation, abnormal cytology, advanced pelvic organ prolapse (stage II or higher), or genital bleeding. After confirming eligibility and obtaining written informed consent, laser therapy was performed.

After removal of vaginal secretions, EMLA cream and lidocaine jelly were applied to the vulvar area to be treated and occluded for approximately 30 min to achieve adequate local anesthesia. The cream was then removed, and fractional CO_2_ laser irradiation was performed using a 360° vaginal probe (MonaLisa Touch®; SmartXide², DEKA, Florence, Italy). Treatment parameters followed the method described by Salvatore et al., with DP mode, power 40 W, dwell time 1,000 µs, and spacing 1,000 µm, delivering energy circumferentially to the entire vaginal wall.

Subsequently, a straight probe was used to irradiate the vulvar vestibule and labia majora. The number and interval of sessions were determined according to symptom severity and patient preference, within the framework of routine clinical practice.

### Outcome measures

The study outcomes included (i) symptom severity scores, (ii) ultrasonographic labia majora thickness, and (iii) patient-reported satisfaction; adverse events were assessed from clinical records. Vaginal length extensibility was measured in routine practice but was not sufficiently complete/standardized for longitudinal analysis and therefore was not included as a prespecified endpoint.

Genitourinary symptoms were assessed using a visual analog scale (VAS), on which patients rated the severity of six GSM-related symptoms—genital itching, vaginal dryness, burning sensation, dyspareunia, vaginal “looseness,” and urinary leakage—from 0 (“no symptoms”) to 10 (“worst imaginable condition”). For longitudinal evaluation, two follow-up windows were defined. The short-term group consisted of women who completed the questionnaire 20–59 days after their first laser session (i.e., within 2 months; *n* = 327), whereas the long-term group comprised those who responded 10–14 months after their final laser session (approximately 1 year; *n* = 94), regardless of the total number of treatments received. When multiple questionnaires were available within the same time window, the latest response was used for analysis. Responders were defined as women with a baseline VAS ≥2 (“eligible”) who achieved a ≥2-point improvement within the corresponding follow-up window. VAS scores were summarized at each assessment window as mean ± standard deviation using symptom-specific complete-case observations available within that window. For longitudinal comparisons, changes were summarized using paired baseline–follow-up VAS values when both were available.

To evaluate structural changes in the vulvar tissue, the maximum thickness of the central portion of the labia majora was measured by ultrasonography before and after laser treatment, using a Voluson P8 system equipped with a 12L-RS linear probe (GE Healthcare). Measurements were obtained at standardized sites in both the laser-only group and the HRT-combination group to enable comparison between treatment modalities. Post-treatment ultrasonography was performed at routine follow-up visits; when paired pre/post values were analyzed, the post-treatment measurement corresponded to the predefined short-term (20–59 days after the first session) or long-term (10–14 months after the final session) windows, depending on availability.

Finally, overall treatment satisfaction was evaluated at follow-up using a self-administered questionnaire. Patients were asked to select one of four response options—“very satisfied,” “satisfied,” “don't know,” or “unchanged/dissatisfied”—and these ratings were used to describe the acceptability and perceived benefit of vaginal and vulvar laser therapy.

### Ethical considerations

The study was conducted in accordance with the Declaration of Helsinki and the International Code of Medical Ethics of the World Medical Association. Written informed consent was obtained from all participants after a full explanation of the nature and purpose of the study. The protocol was approved by the Ethics Committee of Kawasaki Medical School (Decision No. 5568-00; approval date February 18, 2022).

### Statistical analysis

Data in the text and tables are presented as mean ± standard deviation; for non-normally distributed variables, medians with interquartile ranges (IQR) are also reported. The distribution of continuous variables was assessed using the Shapiro–Wilk test.

The Wilcoxon signed-rank test was used to evaluate the statistical significance of paired, non-normally distributed continuous variables before and after treatment. Spearman's correlation analysis was applied to describe relationships between continuous variables. For labia majora thickness, a Mann–Whitney *U* test was used in the laser-only group (where normality was not confirmed), and a paired *t*-test was used in the HRT-combination group (where pre- and post-treatment values were normally distributed).

Statistical analyses were performed using IBM SPSS Statistics for Windows, version 23.0 (IBM Corp., Armonk, NY, USA). A two-sided *p*-value < 0.05 was considered statistically significant.

## Results

### Study flow and follow-up availability

A total of 826 women underwent 2,129 fractional CO_2_ laser sessions during the study period, yielding 7,008 clinical records. Short-term follow-up data (20–59 days after the first session) were available for 327 women, and long-term follow-up data (10–14 months after the final session) were available for 94 women (Figure 1). For symptom-specific analyses, only women with both baseline and follow-up VAS scores were included for each symptom; therefore, the number of paired observations differed by symptom and time window (Table 1).

**Table 1:** Symptom improvement from baseline (median and responder rate).

Window	Symptom	*N*	Median improvement (VAS)	IQR 25%	IQR 75%	Responder rate (≥2 VAS), %	Responders/Eligible
Short-term (20–59 d after first session)	Itching	502	0	0.0	2.0	71.8	158/220
Short-term (20–59 d after first session)	Dryness	503	2	0.0	4.0	77.0	255/331
Short-term (20–59 d after first session)	Burning	492	0	0.0	1.0	76.0	92/121
Short-term (20–59 d after first session)	Dyspareunia	383	1	0.0	4.0	77.5	179/231
Short-term (20–59 d after first session)	Looseness	492	1	0.0	3.0	69.1	208/301
Short-term (20–59 d after first session)	Urinary leakage	504	1	0.0	3.0	73.4	224/305
Long-term (10–14 mo after last session)	Itching	140	0	0.0	2.0	78.3	47/60
Long-term (10–14 mo after last session)	Dryness	140	1	0.0	3.0	76.8	63/82
Long-term (10–14 mo after last session)	Burning	138	0	0.0	0.0	81.8	18/22
Long-term (10–14 mo after last session)	Dyspareunia	96	2	0.0	4.0	88.3	53/60
Long-term (10–14 mo after last session)	Looseness	137	2	0.0	4.0	81.6	71/87
Long-term (10–14 mo after last session)	Urinary leakage	140	1	0.0	4.0	80.2	69/86

### Baseline characteristics

During the 8-year study period, 826 women underwent a total of 2,129 sessions of fractional CO_2_ vaginal and vulvar laser therapy. The mean age at first treatment was 61.9 ± 10.2 years (range, 29–87). Age distribution was as follows: 20 s, 1 (0.1%); 30 s, 14 (1.7%); 40 s, 71 (8.6%); 50 s, 197 (23.9%); 60 s, 296 (35.9%); 70 s, 209 (25.3%); and ≥80 years, 37 (4.5%) (Table 2).

**Table 2A:** Baseline characteristics (overall).

Characteristic	Value
Patients, *n*	826
Age at first treatment, mean ± SD (years)	61.9 ± 10.5
Study period	Jan 2016–Dec 2023

**Table 2B:** Age distribution at first treatment.

Age decade	*n*
20 s	1
30 s	14
40 s	65
50 s	272
60 s	296
70 s	131
80 s	38
90 s	8

**Table 2C:** Distribution of total laser sessions per patient.

Total sessions per patient	*n*
1	263
2	295
3	123
4–5	70
6–9	54
≥10	20

**Table 2D:** Baseline symptom burden (VAS).

Symptom	*N* with baseline recorded	Prevalence (VAS > 0), *n* (%)	Mean ± SD	Median (IQR)
Itching	822	439 (53.4)	2.04 ± 2.68	1 (0–3)
Dryness	820	597 (72.8)	3.50 ± 3.11	3 (0–6)
Burning	819	241 (29.4)	1.21 ± 2.39	0 (0–1)
Dyspareunia	739	501 (67.8)	4.14 ± 3.80	3 (0–8)
Looseness	818	554 (67.7)	3.40 ± 3.14	3 (0–6)
Urinary leakage	822	591 (71.9)	3.32 ± 3.11	3 (0–6)

The number of laser sessions per patient was one in 320 (38.8%), two in 218 (26.4%), three in 103 (12.5%), four in 55 (6.7%), five in 39 (4.7%), six in 30 (3.6%), seven in 19 (2.3%), eight in 13 (1.6%), nine in 11 (1.3%), and ≥10 sessions in 18 (2.2%) (Table 2).

Before treatment, the most frequently reported subjective symptom (multiple responses allowed) was vaginal dryness in 596 women (72.2%), followed by urinary leakage in 590 (71.4%), vaginal looseness in 554 (67.1%), dyspareunia in 494 (59.8%), genital itching in 437 (52.9%), and burning sensation in 240 (29.1%). Although isolated genital symptoms were more than six times as common as isolated urinary symptoms, the most frequent pattern was a combination of both genital and urinary complaints, observed in 560 women (26.4%).

### Changes in VAS scores over time

**Figure 2:**
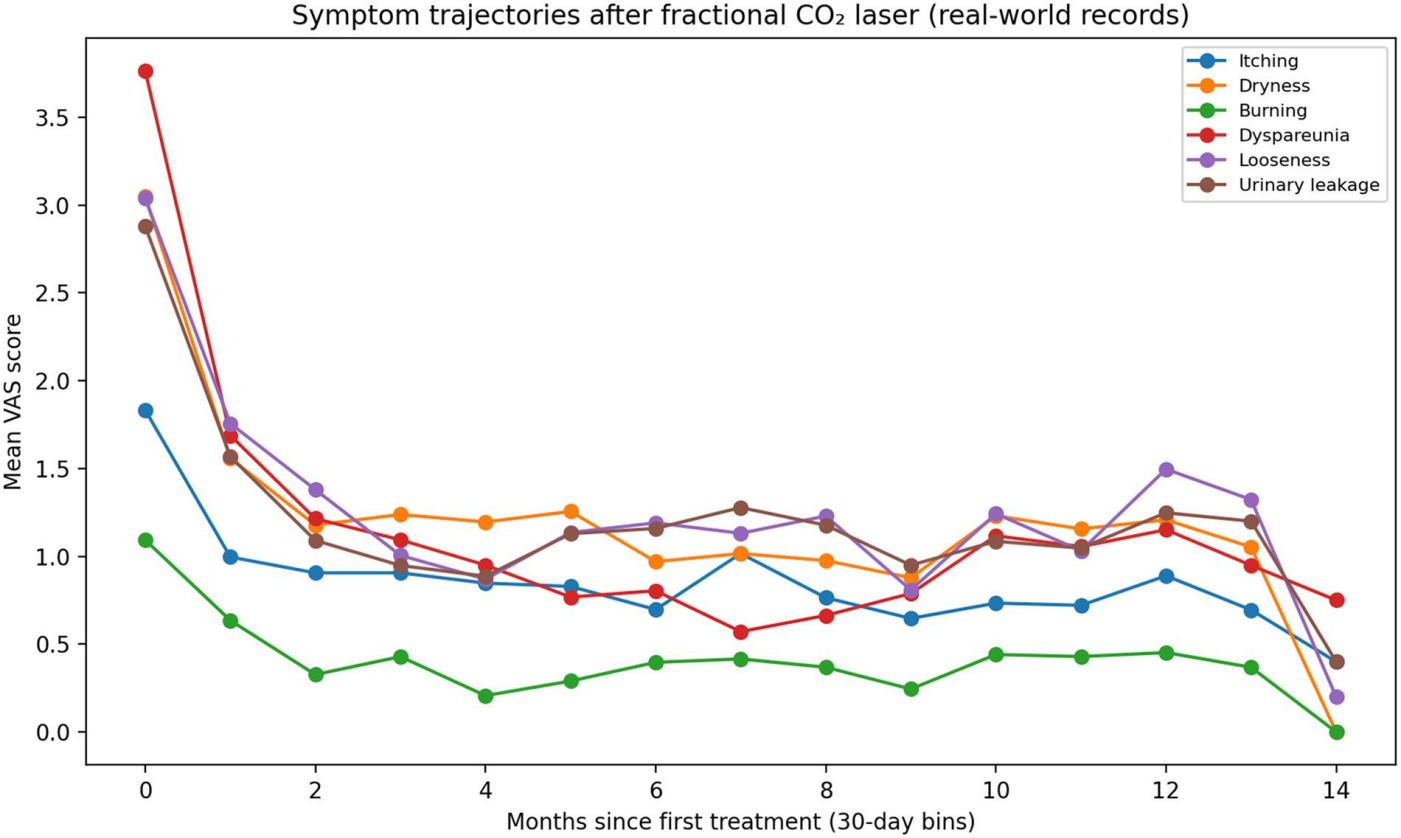
Symptom trajectories after fractional CO_2_ laser therapy. Mean VAS scores for six symptoms (itching, dryness, burning, dyspareunia, vaginal looseness, urinary leakage) plotted over time since the first laser session. Time was grouped into 30-day bins (Month 0 = 0–29 days, Month 1 = 30–59 days, … up to Month 14). Each point represents the mean VAS score within a bin calculated from all available real-world records. Lower scores indicate less severe symptoms.

Symptom score changes were assessed using VAS scores (0–10) for six genitourinary symptoms (Table 1; Figure 2). In the short-term evaluation (20–59 days after the first laser session), median improvements were observed across all symptoms: itching 2, dryness 2, burning 1, dyspareunia 2, looseness 1, and urinary leakage 1. Responder rates (defined as a ≥2-point VAS reduction among women with baseline VAS ≥2) varied by symptom: dyspareunia 81.5%, itching 67.9%, dryness 64.6%, burning 63.9%, looseness 62.0%, and urinary leakage 57.3% (Table 1).

In the long-term evaluation (10–14 months after the final session), improvements persisted but showed symptom-specific attenuation. Dyspareunia remained the most durable outcome (median change 2; responder rate 88.0%). In contrast, dryness (median change 1; responder rate 67.2%) and urinary leakage (median change 1; responder rate 69.1%) showed smaller sustained effects, whereas itching (median change 1), burning (median change 1), and looseness (median change 1) demonstrated modest long-term improvements (Table 1; Figure 2). Overall, these data indicated distinct “decay dynamics,” with dyspareunia showing the most stable long-term benefit.

### Responder rates (symptom-specific; short-term vs. long-term)

Short-term responder rates ranged from 57.3% (urinary leakage) to 81.5% (dyspareunia). Long-term responder rates ranged from 67.2% (dryness) to 88.0% (dyspareunia) (Table 1). Across symptoms, the direction of effect was consistent (improvement), but the magnitude and durability differed, supporting symptom-specific trajectories rather than a uniform “global” response.

### Dose–response relationship (number of sessions and symptom improvement)

A dose–response analysis was performed in the short-term window for dyspareunia, dryness, and urinary leakage (Table 3). For dyspareunia, median VAS improvement increased with greater treatment exposure: 1 session (median change 1), 2 sessions (2), 3 sessions (3), 4 sessions (3), and ≥5 sessions (4). Responder rates also increased from 74.3% (1 session) to 93.3% (≥5 sessions) (Table 3). For dryness, median improvement rose from 2 (1 session) to 3 (≥5 sessions), with responder rates increasing from 62.3% to 73.9%. For urinary leakage, the dose–response was smaller but still graded: median change 1 across categories, while responder rates increased from 54.1% (1 session) to 71.6% (≥5 sessions) (Table 3). These findings indicate an association between greater treatment exposure and higher responder rates, but should not be interpreted as a causal dose–response effect because baseline severity, patient motivation, and follow-up engagement may differ across session-number categories.

**Table 3:** Dose–response (short-term): responder rate by total sessions for key symptoms.

Symptom	Total sessions	*N* (with baseline + FU)	Eligible (baseline ≥2), *n*	Responders (≥2), *n*	Responder rate, %	Median improvement (VAS)
Dyspareunia	1	30	18	12	66.7	0.50
Dyspareunia	2	179	111	84	75.7	1
Dyspareunia	3	77	46	39	84.8	2
Dyspareunia	≥4	97	56	44	78.6	1
Dryness	1	49	27	15	55.6	0
Dryness	2	227	145	119	82.1	2
Dryness	3	106	76	53	69.7	1.50
Dryness	≥4	121	83	68	81.9	2
Urinary leakage	1	49	32	25	78.1	2
Urinary leakage	2	227	127	90	70.9	1
Urinary leakage	3	106	67	44	65.7	1
Urinary leakage	≥4	122	79	65	82.3	2

### Trajectory patterns

Figure 2 summarizes symptom trajectories from baseline to follow-up, demonstrating rapid short-term symptom relief across domains and symptom-specific long-term persistence, with dyspareunia showing the greatest sustained separation from baseline while dryness and urinary leakage showing partial attenuation over time. The number of observations contributing to each time bin is provided in the figure/legend to reflect real-world record aggregation and missingness.

### Patient satisfaction and safety

**Figure 3:**
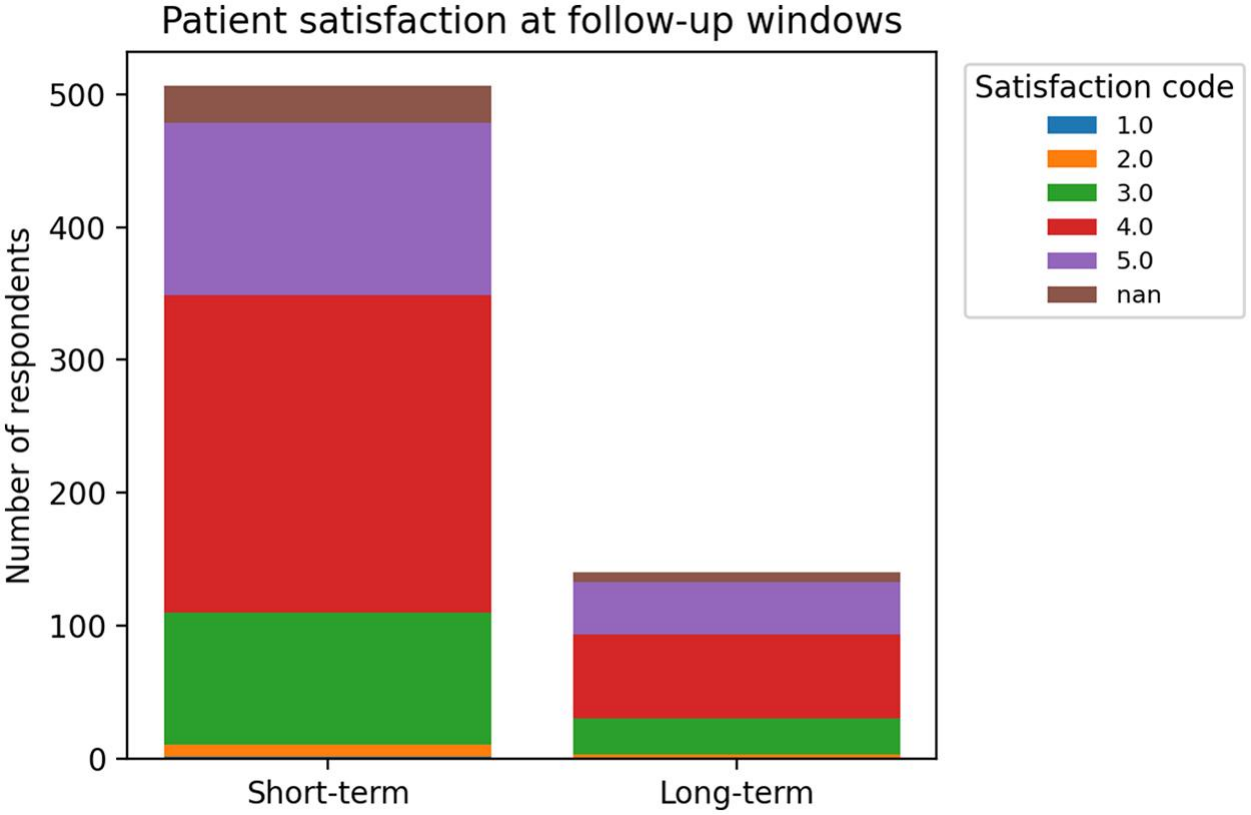
Patient satisfaction after fractional CO_2_ laser therapy. Patient-reported satisfaction was recorded using the original clinic codes without recoding (Code 1–5), with lower codes indicating greater satisfaction (Code 1 = very satisfied; Code 2 = satisfied; Code 3 = don't know; Code 4 = unchanged; Code 5 = dissatisfied). NaN indicates missing or unrecorded satisfaction responses (e.g., questionnaire not completed or item left blank) and these cases were excluded from percentage calculations but are shown to indicate data completeness. NaN, not available.

Patient-reported satisfaction with treatment was high. At both short-term and long-term evaluations, more than 70% of women reported being “very satisfied” or “satisfied” with the laser therapy, indicating high patient-reported acceptability among respondents (Figure 3). Satisfaction was visualized using the original satisfaction codes in the dataset, allowing direct comparison of short-term and long-term distributions without recoding (Figure 3).

Across all 2,129 laser sessions, no serious adverse events, including burns or long-term complications, were observed. Some patients who reported dissatisfaction did so in the context of intercurrent conditions unrelated to GSM (such as Candida vaginitis or vulvar herpes) during follow-up; none of these complaints were attributed to side effects of the laser procedure itself. Because adverse events were captured retrospectively from clinical records, the absence of serious events should be interpreted cautiously and does not replace prospective systematic safety assessment.

## Discussion

Genitourinary syndrome of menopause (GSM) is now understood as a chronic, progressive condition involving not only thinning of the vaginal epithelium but also stromal rarefaction, reduced vascularity, and altered neurosensory signaling across the genital and lower urinary tract, driven primarily by estrogen deficiency (1, 16, 17). These multilayered pathophysiologic changes help explain the wide spectrum of GSM manifestations and the variable response of different symptom domains to a given therapy. In this large real-world cohort of 826 women undergoing 2,129 sessions of fractional CO_2_ laser therapy, we observed distinct symptom-specific trajectories and ultrasonographic changes in vulvar soft tissue thickness. Importantly, our data further illustrate the practical gap between guideline-recommended GSM therapies and real-world care in settings where approved local hormonal options are limited or underutilized, which has contributed to interest in device-based, nonhormonal approaches (1, 5).

One of the most striking observations was the disproportionately robust and durable improvement in dyspareunia compared with other symptoms. Dyspareunia is closely linked to deeper vaginal wall biomechanics—collagen content, elastin architecture, and distensibility—rather than superficial lubrication alone (16, 17). Fractional CO_2_ laser creates controlled microablative thermal injury that stimulates wound-healing cascades, including fibroblast activation, collagen remodeling, and neovascularization in the lamina propria and subepithelial stroma, potentially leading to sustained modifications in tissue structure and mechanical properties (8). This “deeper-tissue” hypothesis provides a coherent mechanistic explanation for why dyspareunia—more than purely mucosal symptoms—showed the most durable benefit in our cohort. Systematic reviews and meta-analyses report improvements in dyspareunia and vaginal health-related outcomes after intravaginal laser, although certainty is often limited by heterogeneity and study design (11, 12, 18–20). Recent society statements and evidence syntheses emphasize that symptom improvement does not automatically equate to disease modification and that durable clinical benefit requires confirmation in well-controlled trials with standardized endpoints and longer follow-up (14, 20).

The significant increase in labia majora thickness documented in more than 80% of women in this study supports the concept of structural change beyond symptom reporting. Previous studies of vaginal laser modalities, including Er:YAG approaches, have described improved vaginal tissue parameters and symptom relief, suggesting that thermal effects can influence atrophic tissue structure (21). Our ultrasonographic findings extend “tissue change” assessment to the vulva, an anatomic domain that is clinically relevant for irritation, friction-related pain, and sexual discomfort, but is less frequently captured in laser studies focused only on the vagina. The finding that women with thinner baseline labial tissue exhibited proportionally greater thickening is biologically plausible, implying that more atrophic tissue may exhibit a larger dynamic range in response to remodeling stimuli. However, imaging changes should be interpreted cautiously: ultrasonography does not provide histologic confirmation, and future work should link objective structural measures with validated patient-reported outcomes and, where feasible, tissue-level correlates (20).

In contrast, improvements in vaginal dryness and urinary leakage, while evident in the short term, partially attenuated over time. This divergence likely reflects fundamental differences in symptom pathophysiology. Vaginal dryness depends heavily on epithelial maturation, mucosal hydration, and local secretory milieu, all of which are strongly estrogen-dependent and may regress relatively quickly when hypoestrogenism persists (1, 16). Urinary leakage is multifactorial (urethral sphincter function, pelvic floor support, and bladder dynamics), and may be less amenable to an intervention primarily targeting vaginal/vulvar mucosa and adjacent stroma. Consistent with this, sham-controlled trials and RCT-focused meta-analyses have reported mixed or modest effects, particularly for urinary outcomes, and have highlighted limitations in certainty (12, 13, 19, 20). Our symptom-specific “decay” pattern (durable dyspareunia improvement with partial attenuation of dryness/urinary leakage) is therefore clinically plausible and aligns with the broader evidence base that different GSM domains may require different maintenance strategies or combination therapy (1, 20).

Beyond mean changes, responder analysis and exposure–response (“dose–response”) patterns can strengthen the clinical interpretability of device-based interventions by translating statistical change into patient-relevant benefit. In pain-related and symptom-intensity outcomes measured on 0–10 scales, a ∼2-point improvement is commonly used as a clinically meaningful threshold, and responder reporting is recommended to complement mean change, especially when placebo/sham responses are substantial (22, 23). In sham-controlled trials of vaginal laser for GSM, between-group differences may be modest despite within-group improvement, highlighting the value of presenting the proportion of patients who achieve meaningful relief and identifying which domains are most likely to respond (12, 13, 20). In our cohort, symptom-specific responder rates and the graded increase in responder frequency with higher numbers of sessions (most pronounced for dyspareunia, and more moderate for dryness and urinary leakage) are consistent with multi-session paradigms used in early prospective studies and larger cohorts, where repeated treatments are intended to reinforce remodeling and symptom control (9, 10). Taken together, these results support a pragmatic implication for real-world counseling and service design: treatment benefit is heterogeneous across symptom domains, and both the probability of response and the need for additional sessions may differ by symptom phenotype—an “analysis → implication” bridge that should be prospectively tested using prespecified responder endpoints and maintenance schedules (20, 24).

A practical implication of these symptom-specific trajectories is the likely need for maintenance therapy. GSM is chronic and typically requires ongoing management; guideline documents emphasize long-term approaches and reassessment rather than one-time interventions (1). In our cohort, the attenuation of dryness and urinary leakage over ∼1 year supports counseling patients that (i) benefits may vary by symptom domain and (ii) repeated sessions or adjunctive therapies may be needed to sustain improvements in mucosa-dependent or multifactorial urinary symptoms. Defining optimal maintenance intervals remains an evidence gap and should be a priority for prospective studies that prespecify maintenance schedules and compare them with standard-of-care therapies (20).

Safety remains central in discussions of energy-based devices for GSM. Professional society statements have recommended caution and have advised that vaginal laser should not be routinely offered outside clinical trials in many settings (14). Regulatory communications have also highlighted potential risks when energy-based devices are marketed for “vaginal rejuvenation” indications without robust evidence (25). In our cohort, no serious adverse events were observed across more than 2,000 sessions, and dissatisfaction was largely associated with intercurrent conditions rather than apparent procedure-related complications. These findings are reassuring for procedural safety in an experienced clinic using standardized protocols and careful pre-treatment evaluation; however, absence of observed serious events in a retrospective cohort does not substitute for long-term, systematically ascertained safety outcomes (20).

This study has several strengths. It is one of the largest single-center cohorts of women treated with fractional CO_2_ vaginal and vulvar laser therapy, providing real-world data across a wide age range. We combined symptom VAS measures with ultrasonographic assessments of vulvar soft-tissue thickness, providing convergent descriptive data on patient-reported symptom trajectories and imaging changes. However, several limitations must be acknowledged. The retrospective observational design precludes causal inference and is susceptible to selection and information bias. There was no sham-treated or non-laser control group, and follow-up was incomplete and not uniform. Symptom assessment relied on VAS scoring rather than fully validated GSM instruments, and objective measures lacked histologic confirmation. Additionally, because this was real-world practice, concomitant treatments and care-seeking behavior may have varied over time, which can influence both responder classification and apparent dose–response patterns. These issues should be addressed prospectively (1, 20).

Despite these limitations, our findings provide descriptive real-world data on symptom trajectories and ultrasonographic changes in a large Japanese cohort. Given the lack of a control/sham group and incomplete follow-up, the results should be considered hypothesis-generating rather than confirmatory evidence of effectiveness (14, 20, 26).

## Data Availability

The raw data supporting the conclusions of this article will be made available by the authors, without undue reservation.
